# Defining the Optimal Microspore Developmental Window for Efficient Anther-Derived Somatic Embryogenesis in Rubber Tree (*Hevea brasiliensis*)

**DOI:** 10.3390/plants15060973

**Published:** 2026-03-21

**Authors:** Yinglian Wu, Naushad Alam, Xing Bao, Suna Peng, Rizhi Wu, Chenrui Gu, Xinran Ou, Haobin Liu, Xiaoyi Wang, Tiandai Huang

**Affiliations:** 1 State Key Laboratory of Tropical Crop Breeding, Rubber Research Institute, Chinese Academy of Tropical Agricultural Sciences/Haikou Key Laboratory of Tropical Plant Seedling Innovation/Key Laboratory of Biology and Genetic Resources of Rubber Tree, Ministry of Agriculture and Rural Affairs/Key Laboratory of Tropical Crop Cultivation Physiology of Hainan Province/State Key Laboratory Cultivation Base, Haikou 571101, China; 2 College of Plant Science and Technology, Huazhong Agricultural University, Wuhan 420010, China; 3 Sanya Research Institute, Chinese Academy of Tropical Agricultural Sciences, Sanya 572025, China; 4 College of Tropical Agriculture and Forestry, Hainan University, Haikou 570228, China

**Keywords:** flower buds, anther-derived callus, cytology, regeneration

## Abstract

Anther-derived somatic embryogenesis is a valuable approach in rubber tree (*Hevea brasiliensis*) breeding; however, its effectiveness is highly influenced by the developmental stage of the microspores. The present investigation focused on male flower buds of the cultivar Reyan 73397 at successive developmental stages to examine the relationship between visible bud characteristics and internal microspore development, assess how microspore developmental stage affects callus induction and somatic embryo formation, and identify the stage with the greatest embryogenic potential. Cytological observations distinguished six well-defined phases of microspore development, spanning from microspore mother cells to fully mature pollen grains, each reliably linked to particular bud diameters, coloration, and anther morphology. Anthers corresponding to each developmental phase were cultured in vitro, and their ability to initiate callus and produce somatic embryos was systematically evaluated. Anthers containing uninucleate microspores exhibited the highest rates of both callus formation and somatic embryogenesis, with the early-uninucleate stage showing the strongest response. This stage consistently matched flower buds measuring 1.42–1.57 mm in transverse diameter and displaying a green to yellowish-green appearance. In contrast, anthers collected at the microspore mother cell and tetrad stages did not produce embryogenic responses. Histological evidence has indicated that both callus and somatic embryos originate from diploid somatic tissues of the anther wall, particularly connective parenchyma cells, rather than from microspores themselves. Based on these findings, a rapid, non-destructive selection method integrating bud diameter, bud color, and sieve-based size separation was developed to identify responsive explants efficiently. Overall, this study defines the optimal developmental window for anther culture in rubber trees, verifies the somatic origin of embryogenic tissues, and provides a practical morphological and cytological basis for improving anther culture efficiency in rubber tree breeding programs.

## 1. Introduction

The rubber tree (*Hevea brasiliensis*) is the world’s primary commercial source of natural rubber and is grown predominantly in Southeast Asia and in West Africa [1]. In China, rubber plant cultivation is restricted in the provinces of Hainan, Yunnan, and Guangdong [2]. China is also the largest consumer of natural rubber; in 2023, its consumption reached 7.001 million metric tons, accounting for 46.03% of global demand. However, more than 80% of this requirement is encountered through imports [3]. With continued economic growth, domestic demand, in response to supply, is expected to continue to grow in the future, emphasizing the urgent need to develop high-yielding rubber tree varieties. *Hevea brasiliensis* is a cross-pollinated, monoecious species that appears with unisexual flowers arranged in racemose inflorescences [4]. Traditional plant breeding relying on manual pollination is time-consuming, labor-intensive, and requires technical expertise [5]. Moreover, the high degree of heterozygosity in rubber tree populations complicates the stable inheritance of elite traits through seed propagation [5]. These constraints have spurred increased interest in plant biotechnology approaches, including tissue culture-mediated regeneration, which exploit cellular totipotency to regenerate whole plants from cultured explants under controlled in vitro conditions [3,5]. Since the 1970s, anther culture has been established as a key technique for somatic embryogenesis and plant regeneration [6,7]. By inducing microspores to develop into haploids followed by chromosome doubling, this technique enables the rapid generation of homozygous plants, thereby significantly improving breeding efficiency [8]. However, in rubber trees (*Hevea brasiliensis*), isolated microspore culture has not yet succeeded in inducing haploid plants [9]. In most plant species, regenerated plants derived from anther culture predominantly originate from anther somatic tissues rather than microspores [10,11,12,13,14]. Despite this limitation, efficient diploid somatic embryogenesis from anthers is highly valuable. The anther provides a microenvironment that supports clonal propagation, variation, gene editing, and germplasm conservation, emphasizing its broad applications in plant biotechnology and breeding. Somatic embryo induction from anther-derived tissues is influenced by multiple factors, among which the developmental stage of microspores is considered particularly important. In haploid induction systems, only microspores at specific stages—such as late uninucleate or binucleate—are competent to respond to inductive signals and redirect their developmental program from the gametophytic pathway to embryogenesis. In most plant species, this critical period coincides with the first pollen mitosis, which typically occurs during the late uninucleate phase [15]. This phase is marked by a prominent central vacuole and a nucleus located at the periphery. It has been recognized as the ideal stage for inducing embryogenesis in numerous species, including carrot (*Daucus carota* var. *sativus*) [16], *Brassica nigra* [17], and almond (*Prunus dulcis*) [18], although there are notable exceptions. For example, the binucleate stage is the most responsive in *Brassica napus* [19], whereas tetrad-stage microspores show developmental plasticity in cassava (*Manihot esculenta*) [20]. However, the regulatory role of the microspore developmental stage in anther-derived somatic embryogenesis in rubber trees remains poorly understood.

Numerous studies on different plant species have reported that the stages of microspore development are intricately linked to external floral characteristics, such as bud size, petal length, and anther color, enabling the indirect, non-invasive determination of these stages [21]. In this study, the microspore developmental stage was only used as a reference for selecting flower buds. The embryogenic calli and plant regeneration were derived from anther wall somatic cells, corresponding to a somatic embryogenesis pathway rather than microspore-derived androgenesis. Bud length has proven to be an effective indicator for identifying the optimal developmental window for anther culture in *Jatropha curcas* and *Quercus suber* [22,23]. In barley (*Hordeum vulgare*), the gap between the flag leaf and the penultimate leaf is a reliable marker for determining the optimal stage of anther development [24]. However, genotype- and environment-dependent variations can compromise the reliability of morphological characteristics alone, emphasizing the need for cytological techniques such as acetocarmine staining or DAPI-based fluorescence microscopy to accurately determine developmental stages. Consequently, cytological observation remains the most reliable method for precise determination of microspore stage, with external traits serving as rapid, preliminary screening tools [25].

Although many efforts have been made to optimize anther-regeneration media for different Para rubber cultivars, few studies have systematically examined the influence of the microspore developmental stage on somatic embryogenesis efficiency in rubber trees [10,26]. To address this knowledge gap, this study examined whether the external morphological features of flower buds and anthers consistently reflect the internal developmental stages of microspores in *H. brasiliensis*. We also assessed the embryogenic potential of anthers containing microspores at different developmental stages to identify the stage that is most favorable for somatic embryo induction. By defining practical morphology-based criteria for selecting highly responsive anthers, this study seeks to improve anther culture protocols and support more efficient rubber tree breeding programs.

## 2. Results

### 2.1. Study of Microspore Development

**Figure 1:**
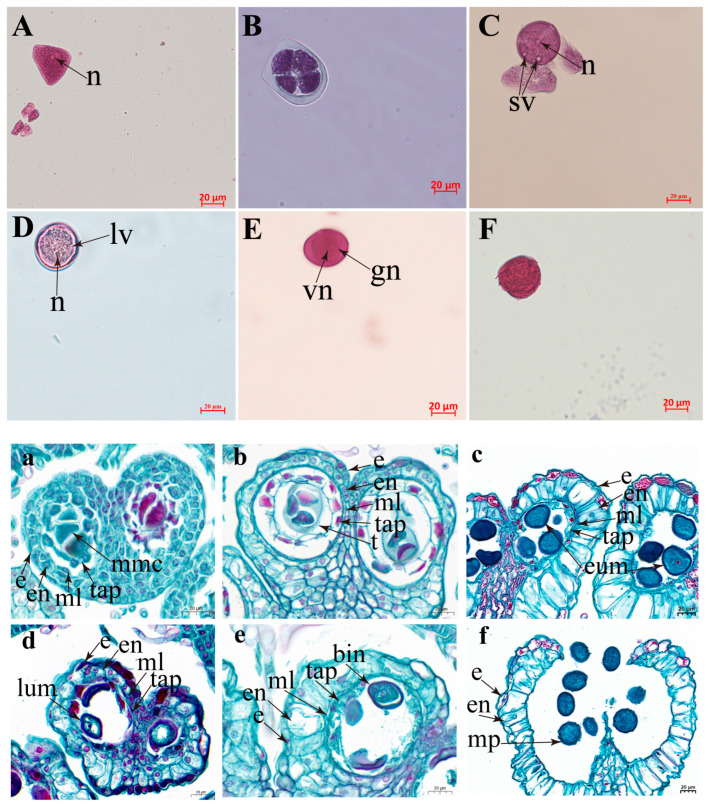
Cytological and histological characterization of microspore development and pollen formation. (**A**,**a**) Microspore mother cell stage; (**B**,**b**) tetrad stage; (**C**,**c**) early uninucleate stage; (**D**,**d**) late uninucleate stage; (**E**,**e**) binucleate stage; and (**F**,**f**) mature pollen stage. Detailed histological observations illustrate the distinct anther wall layers and cellular differentiation: epidermis (e), endothecium (en), middle layer (ml), and tapetum (tap). Developmental stages are indicated as follows: microspore mother cell (mmc), tetrad (t), early uninucleate microspore (eum), late uninucleate microspore (lum), binucleate microspore (bin), and mature pollen grain (mp). Cytological characteristics during microspore development are labeled as follows: nucleus (n), small vacuoles (sv), large vacuole (lv), vegetative nucleus (vn), generative nucleus (gn). Red and black scale bars = 20 μm.

The morphological characteristics of the developmental stages of microspores of *Hevea* varieties (Reyan 73397) are presented in Figure 1. Cytological observations indicated that the microspore development follows six developmental stages. Microscopic observation revealed a single large nucleus and an irregular shape, characteristic of the microspore mother cell stage (Figure 1A,a). Following meiotic division, the microspore assumed a tetrahedral arrangement and was enclosed by a callose wall that is characteristic of the tetrad stage (Figure 1B,b). Subsequent degradation of the callose wall released individual microspores. These free unicellular microspores had a large, clearly stained nucleus in the cell center, representing the early uninucleate stage (Figure 1C,c). During development, we observed a vacuole forming in the cell, and the nucleus migrated to the cell edge, indicating the late uninucleate stage (Figure 1D,d). Microspores with two nuclei were found and regarded as the binucleate stage (Figure 1E,e). The mature pollen stage is characterized by well-defined germination pores (Figure 1F,f).

**Figure 2:**
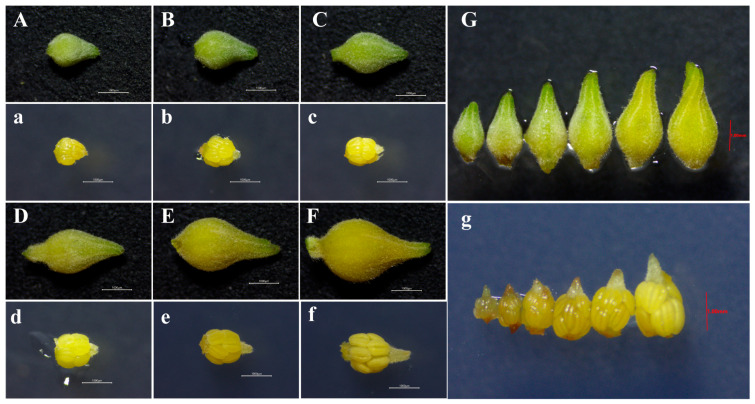
Morphological features of floral buds and anthers at successive developmental stages. (**A**,**a**) Floral bud and anthers at the microspore mother cell stage. (**B**,**b**) Floral bud and anthers at the tetrad stage. (**C**,**c**) Floral bud and anthers at the early uninucleate stage. (**D**,**d**) Floral bud and anthers at the late uninucleate stage. (**E**,**e**) Floral bud and anthers at the binucleate stage. (**F**,**f**) Floral bud and anthers at the mature pollen stage. (**G**,**g**) Progressive development of the flower in relation to clearly defined anther developmental stages. Scale bars represent 1000 μm and 1.00 mm, respectively.

The size and morphological features of flower buds corresponding to different microspore developmental stages were examined, with variations recorded in bud length, width, and color. Flower buds with a length of ~1.68 mm and a width of ~1.14 mm, light green in color, with the calyx tightly enclosing the corolla, and anthers with a tender, translucent texture, were revealed as the microspore mother cell stage. The tetrad stage was characterized by flower buds measuring ~2.02 mm in length and ~1.25 mm in width, light green in color, with the calyx tightly enclosing the corolla, and anthers turning milky white and gel-like. At the early uninucleate stage, the flower bud is ~2.34 mm in length and ~1.42 mm in width, green, with the calyx tightly enclosing the corolla, and the anthers are creamy yellow with a slightly firm texture. The late uninucleate stage is characterized by buds with a length of ~2.74 mm and a width of ~1.57 mm, a greenish-yellow colour, with the calyx tightly enclosing the corolla, and anthers that are pale yellow and flexible. The binucleate stage is characterized by buds with a length of ~3.04 mm and a width of ~1.65 mm, yellow with a yellowish-green colour, the calyx enclosure starting to loosen, and anthers light yellow and semi-rigid. The mature pollen stage was associated with buds approximately 3.46 mm in length and 1.97 mm in width, yellow in color, with a slightly open calyx and yellowish-white anthers that had not undergone complete sclerosis (Table 1; Figure 2).

Significant differences were recorded in the length of flower buds at different developmental stages. Bud width helped distinguish most stages but failed to differentiate adjacent stages, such as the early and late uninucleate stages. Flower bud color also allowed differentiation of most stages, but could not distinguish adjacent developmental stages. The flower buds were covered with short villi throughout development, and these villi showed no apparent correlation with the microspore developmental stages (Table 1, Figure 2). The study also found that asynchrony in microspore development occurred among different anthers within the same inflorescence or among different locules within the same anther. At the same time, the primary developmental stage was dominant in a single anther, and the developmental stages were relatively consistent within the same locule.

### 2.2. Callus Induction

**Figure 3:**
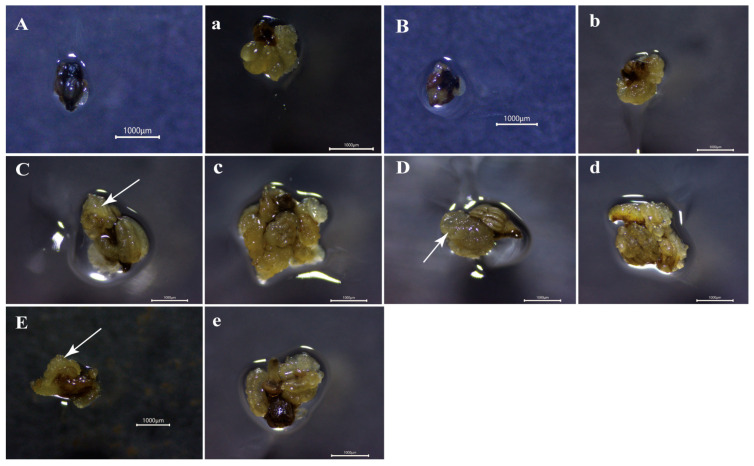
Anther calli at different developmental stages after 21 and 28 days of culture. (**A**,**B**) Non-responsive anthers and anther-derived calli at 21 d of culture. (**C**–**E**) Responsive anthers and anther-derived calli at 21 d of culture. (**a**–**e**) Responsive anthers and anther-derived calli at 28 d of culture. White arrow indicates the callus derived from the anther explant in response to callus induction. Scale bars represent 1000 μm.

Callus induction from anthers was significantly associated with the microspore developmental stage (Table 2). Induction efficiency increased with anther maturation, peaking at the early-uninucleate stage (100%), followed by the late uninucleate (98.81%) and binucleate (97.62%) stages, after which it declined. These three stages were classified into the same statistical group (group a, Table 2)**.** However, all yielded significantly higher induction rates than the tetrad (32.14%, group b) and microspore mother cell (23.81%, group c) stages. In addition to induction rates, the callus quality evaluated using a quantitative scoring system varied significantly among stages, with scores ranked as follows: early uninucleate > late uninucleate > binucleate > tetrad > microspore mother cell. Calli obtained from the three peak stages showed superior quality compared with those from earlier stages, although they did not differ significantly from each other. Furthermore, callus initiation in the responsive stages (early to late uninucleate and binucleate) occurred within three weeks (Figure 3C–E). In contrast, the tetrad and microspore mother cell stages required approximately four weeks (Figure 3A,B). Overall, these findings indicate that the early uninucleate-to-binucleate stages represent the most favorable window for efficient anther-derived callus induction in the rubber tree.

### 2.3. Somatic Embryogenesis and Plant Regeneration

**Figure 4:**
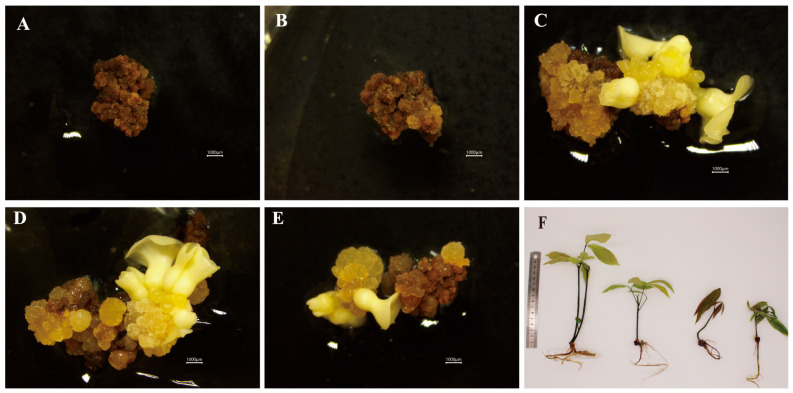
Assessment of somatic embryo conversion efficiency 50 days after callus inoculation and subsequent plantlet regeneration from anther-derived calli at different developmental stages. (**A**) Callus induced from anthers at the microspore mother cell stage, showing no evidence of embryo formation. (**B**) Callus derived from tetrad-stage anthers with no embryogenic response. (**C**) Callus obtained from early uninucleate-stage anthers exhibiting somatic embryo development. (**D**) Callus from late uninucleate-stage anthers displaying somatic embryos. (**E**) Callus derived from binucleate-stage anthers with evident somatic embryo formation. (**F**) Regenerated plantlet originating from a somatic embryo produced by callus induced at the early uninucleate stage. Scale bar: 15 cm.

Somatic embryogenesis efficiency was significantly affected by the developmental stage of microspores in the anthers (Table 3). The highest frequency of embryogenic callus rate was observed at the early uninucleate stage (29.76%), after which induction efficiency declined. The rate at this peak stage was significantly higher than that at the binucleate (12.2%), microspore mother cell (0%), and tetrad (0%) stages. The late uninucleate stage showed an intermediate induction rate (19.28%) that did not differ significantly from either the early uninucleate or the binucleate stages. No somatic embryos were obtained from anthers at the microspore mother cell or tetrad stages (Figure 4A,B). Among the responsive stages, the early uninucleate, late uninucleate, and binucleate stages produced eight, six, and three cotyledonary embryos, respectively (Figure 4C–E). Subsequent plant regeneration from these embryos produced five plantlets in total: four from the early uninucleate stage (50% regeneration rate) and one from the late uninucleate stage (16.67%). No plants regenerated were achieved from embryos induced at the binucleate stage (Figure 4F). Consequently, the early uninucleate and late uninucleate stages are identified as the optimal developmental phase for both somatic embryo induction and plant regeneration from anther-derived calli in the rubber tree.

### 2.4. Microscopic Study on Callus Induction and Embryo Formation with the Developmental Stage of the Anther

**Figure 5:**
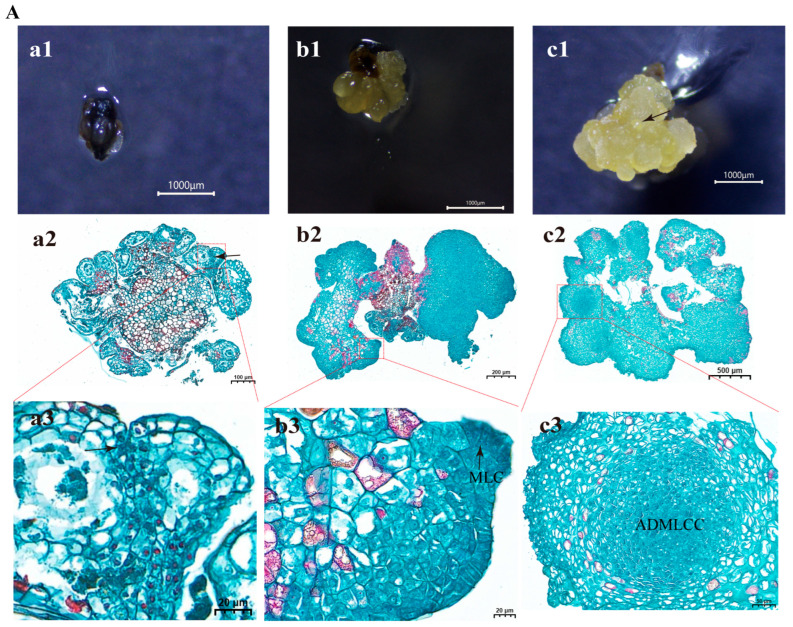

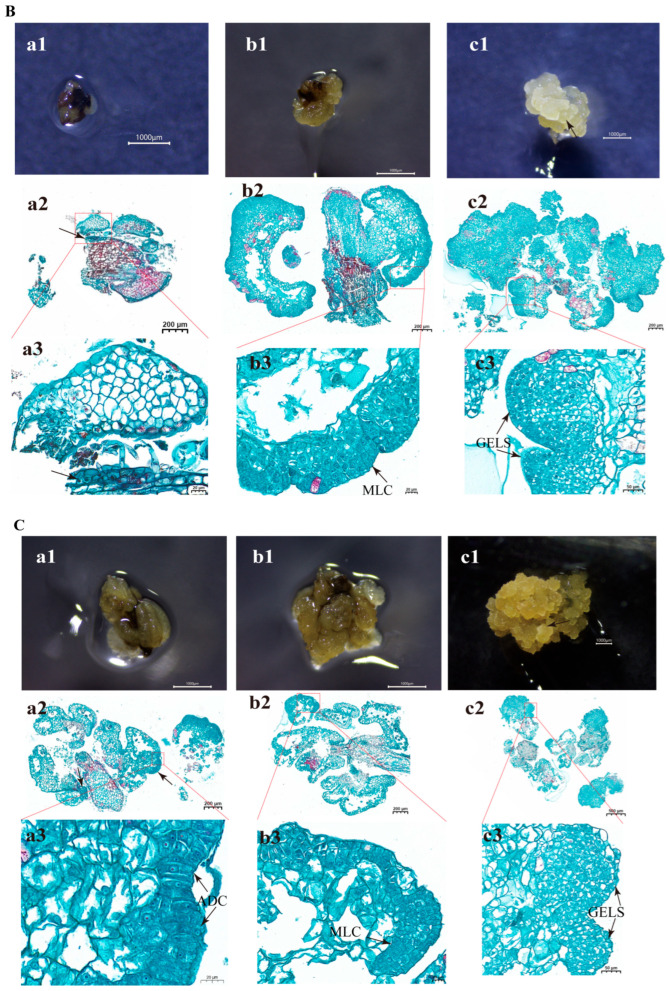

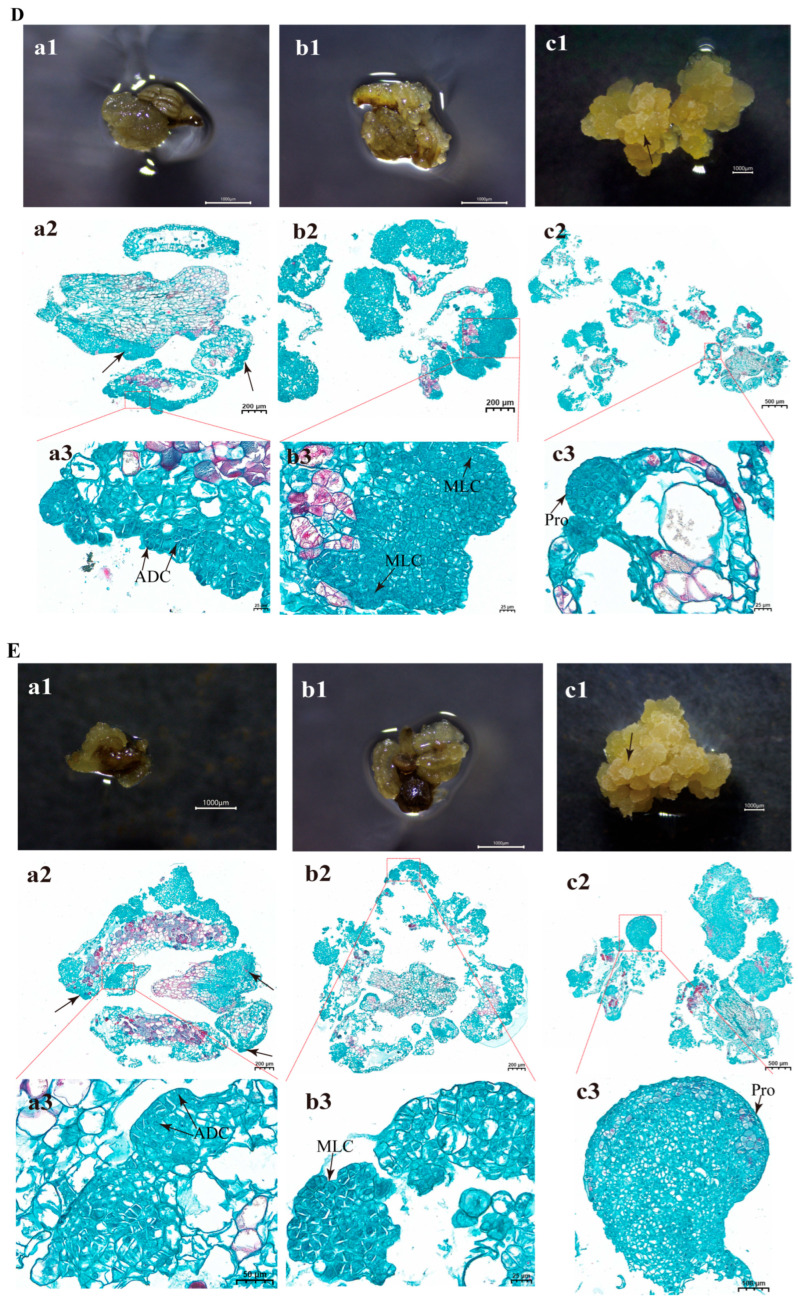
Morphological and histological changes during anther culture at different developmental stages and culture durations (21, 28, and 35 days). (**A**–**E**): Anthers collected at five microspore developmental stages, microspore mother cell (MMC), tetrad, early uninucleate, late uninucleate, and binucleate, were cultured and evaluated after 21, 28, and 35 days. For each stage, the upper row (e.g., **a1**–**c1**) shows the external morphology of cultured anthers, while the middle and lower rows (e.g., **a2**–**c2** and **a3**–**c3**) present corresponding histological sections at low and higher magnifications, respectively. At 21 days, limited callus formation and early cellular reorganization were observed, with structural features of the original anther tissues still visible in most stages. By 28 days, progressive tissue proliferation and callus initiation were evident, particularly in the early and late uninucleate stages, where anther-derived divisions and cellular enlargement were more pronounced. After 35 days of culture, extensive callus formation and tissue disintegration of the original anther wall layers were apparent, especially in the uninucleate and binucleate stages, indicating enhanced embryogenic or callogenic response. Red boxed areas indicate regions selected for magnified observation, and arrows highlight areas of active cell division, tissue proliferation, or callus development. MLC: meristem-like cell; ADMLCC: actively dividing meristem-like cell cluster; ADC: actively dividing cell; GELS: globular embryo-like structure; Pro: protoderm. Scale bars represent 1000 µm (macroscopic images) unless otherwise indicated.

After 21 days of culture on callus induction medium, anthers at the early and late uninucleate and binucleate stages exhibited conspicuous locule swelling when examined under a stereomicroscope, and most had dedifferentiated into well-defined callus masses. Histological analysis of these responsive anthers showed that the callus was composed of densely packed cells with large nuclei and dense cytoplasm that stained intensely, indicative of meristematic activity. Active cell division was observed across multiple somatic tissues, including the epidermis, endothecium, connective tissue, and filament (Figure 5C–E (panel a2), black arrows). During the early phase of dedifferentiation, epidermal cells showed active cell division (Figure 5C,D (panel a3), black arrows). In contrast, anthers at the microspore mother cell and tetrad stages did not exhibit noticeable swelling at this time point (Figure 5A,B (panel a1)), and histological sections displayed only limited mitotic activity, confined mainly to the connective tissue and filament (Figure 5A,B (panel a2), black arrows).

By 28 days of culture, extensive callus formation was observed in anthers at the early and late uninucleate and binucleate stages (Figure 5C–E (panel b2)). While anthers at the microspore mother cell and tetrad stages also exhibited apparent swelling at this stage (Figure 5A,B (panel b2)), their overall developmental progression remained less advanced. Histological observations confirmed active cell division across all stages at 28 days, with sections showing densely arranged, deeply stained cells characterized by small size, large nuclei, and dense cytoplasm (Figure 5A–E (panel b3), black arrows).

After 35 days of culture, stereomicroscopic examination revealed the presence of spherical and pro-embryonic structures with smooth surfaces on the callus (Figure 5A–E (panel c1), black arrows). Histological analysis indicated two distinct levels of organization within these structures. A true globular embryo is spherical, characterized by small, uniform cells with large nuclei and dense cytoplasm that are tightly packed, and possesses a differentiated protoderm. Calli derived from both the late uninucleate and binucleate stages successfully developed into immature globular embryos, which exhibited these canonical features, including symmetrical cell divisions, deep staining, and the formation of a well-defined protoderm (Figure 5D,E (panel c3), black arrows). Calli derived from the tetrad and early uninucleate stages exhibited meristematic cellular characteristics and were morphologically similar to globular embryo-like structures (GELS) (Figure 5B,C (panel c3), black arrows). Morphologically, they were roughly spherical, with typical meristematic characteristics, showing superficial similarities to globular embryos. However, neither structure developed a differentiated protoderm—a key histological hallmark of true globular embryos. Therefore, these structures were identified as non-embryonic globular cell aggregates rather than true globular embryos. Callus derived from anthers at the microspore mother cell stage contained internally formed clusters of densely stained, actively dividing meristem-like cells (Figure 5A (panel c3)).

Collectively, these observations suggest that anther-derived callus in the rubber tree is primarily from somatic tissues of the anther wall, including the epidermis, endothecium, connective tissue, and filament. The initial microspore developmental stage strongly influenced the progression of callus development. In anthers at the uninucleate to binucleate stages, dedifferentiation and callus cluster formation became evident by approximately 21 days, followed by rapid proliferation by 28 days, and the onset of morphologically distinct early embryogenic organization (spherical or pro-embryonic structures) by 35 days. In contrast, anthers at the microspore mother cell and tetrad stages initiated dedifferentiation roughly one week later, indicating a pronounced developmental delay.

## 3. Discussion

The success of anther culture in woody perennial crops is strongly influenced by the physiological status of the donor tissue. In this study, we investigated the relationships among microspore development, external flower–bud morphology, and anther-derived callus induction and somatic embryogenesis in *Hevea brasiliensis* cv. Reyan 73397. Our results demonstrate that embryos originate from somatic tissues of the anther wall rather than from microspores. Accordingly, the developmental stage of microspores should be interpreted as an indicator of overall anther developmental status, rather than as a direct determinant of embryogenic competence.

Anthers containing microspores at the early- to late-uninucleate stages exhibited the highest morphogenic responses; however, this association likely reflects the optimal physiological condition of the surrounding somatic tissues at these stages. Previous studies have highlighted the importance of the microspore developmental stage in haploid and androgenic systems, including rubber tree anther culture [8]. In contrast, our findings suggest that, under the present conditions, embryogenesis follows a somatic pathway, and therefore, the role of the microspore stage is indirect.

The identification of suitable explant stages remains challenging, as conventional methods such as squash preparations and paraffin sectioning are destructive and time-consuming [27]. Establishing correlations between microspore developmental stages and external bud morphology provides a practical, non-destructive approach for selecting responsive explants. Similar relationships have been reported across diverse plant species, where bud size or morphological traits serve as reliable indicators of internal developmental phases [28]. These morphological markers enhance the reproducibility and efficiency of culture systems by enabling the selection of explants at physiologically favorable stages. For example, bud transverse diameter in *Eucalyptus* [29], the transverse-to-longitudinal diameter ratio in *Camellia oleifera* [25], sepal coverage in chili pepper (*Capsicum annuum* L.) [30], bud length in *Pisum sativum* L. and *Pisum sativum* [28,31], and heritable bud size in Chinese kale (*Brassica alboglabra* Bailey) and false pakchoi (*Brassica campestris* L. ssp. *Chinensis* var. *utilis* Tsen et Lee) [32] have all been successfully used to predict key developmental windows. In the present study, such morphological indicators are better interpreted as proxies for optimal anther tissue status rather than as direct predictors of microspore-derived embryogenesis.

In our findings, cytological examinations identified six successive stages of microspore development. Each stage was found to be linked to specific traits in the flower buds, including size, color, and anther morphology. Among the visible characters, bud length appeared to be a useful indicator for distinguishing microspore developmental stages; however, this conclusion is based on descriptive observations rather than statistically validated analyses. Bud width and coloration were less reliable for distinguishing closely related developmental stages, such as the early and late uninucleate stages [33]. Similar observations have been reported in other plant species, where bud length serves only as a rapid but approximate indicator of the microspore developmental stage [34]. Because microspore development occurs within the anther locules, cytological verification remains essential for accurate stage identification in experimental studies. In *Hevea brasiliensis*, each anther locule generally contained a single predominant developmental stage, indicating a relatively synchronized pattern of microspore development. However, buds containing mixed developmental stages showed improved uniformity in in vitro responses. These findings highlight the importance of combining external morphological traits with cytological examination to achieve precise stage determination, particularly for large-scale anther culture applications.

The anther and microspore development of the rubber tree have been well characterized. The anther wall consists of four layers, including an endothecium, a transient middle layer, and a secretory tapetum that degenerates by the three-cell pollen stage [35]. In contrast to normal development observed in seedlings, commercial cultivars such as RRIM600 and GT1 exhibit extensive post-tetrad microspore abortion, with GT1 additionally showing abnormal tapetal enlargement and vacuolation [35]. Developmental asynchrony is particularly pronounced in RRIM600, occurring both among buds and within anther locules. During the pachytene-to-tetrad transition, buds measure approximately 1.63–1.83 mm in length and 1.12–1.21 mm in width, with minimal dimensional variation, limiting the reliability of visual staging. To address this limitation, a practical morphological indicator has therefore been established based on the panicle’s primary subsidiary bud unit. When the primary bud is entirely separated and about 1.5 times the size of its subsidiaries, it typically corresponds to the uninucleate microspore stage [36].

Consistent with these reports, the present study observed marked asynchrony in microspore development among different buds of the same inflorescence and among locules within a single bud of the cultivar ‘Reyan 73397’. Xie [37] evaluated the relationships between bud dimensions, bud color, anther color, and microspore developmental stages in seedling trees and the cultivars RRIM600 and PB86. Flower bud sizes differed among different genotypes at the same stage of development; bud and anther color remained conserved, and color was found to be a reliable indicator across the genetic level. All three genotypes exhibited light-yellow buds and anthers at the uninucleate stage [37]. However, bud length and width remained effective for stage identification within a given genotype [37]. Previously, similar results were documented in ‘Reyan 73397’ [38]. Our findings corroborate the utility of bud length as a primary morphological marker for microspore staging in this cultivar. Despite its usefulness, dependence on bud length alone proved insufficient for efficient large-scale screening. A study found that early- and late-uninucleate stages in ‘Reyan 73397’ gave the highest callus induction and somatic embryogenesis rates. Although transverse bud diameter differed significantly between the uninucleate stages and the microspore mother cell, tetrad, and mature pollen stages, no significant difference was observed between the late-uninucleate and binucleate stages. However, these two developmental stages can be distinguished by bud color: the late uninucleate stage shows a greenish-yellow color, and the binucleate stage appears yellowish-green. Therefore, dimensional measurements and color-based traits allow more accurate and efficient developmental staging. Similarly, based on transverse bud diameter for bud selection, Li et al. [39] also reported this. This method was found to be based on successive sieving of buds using different mesh sizes of 10 (2.0 mm), 16 (1.25 mm), 12 (1.6 mm), and 14 (1.43 mm) to separate buds within a defined size range. The selected buds show transverse diameters of 1.3–1.5 mm and longitudinal diameters of 2.3–3.2 mm. In cultivars ‘Reken 628’, ‘Yunyan 7346’, and ‘Yunyan 73-477’, this approach resulted in anther callus induction and somatic embryogenesis efficiencies compared to manual selection [39]. In the present study, uninucleate-stage buds of ‘Reyan 73397’ measured 1.42–1.57 mm in transverse diameter and 2.34–2.74 mm in length, with coloration ranging from green to greenish-yellow. Collectively, these findings, combining bud size, color, and cytological assessment, provide reliable and practical criteria for selecting the optimal microspore stage in the rubber tree anther culture.

Our results demonstrated that anthers containing early or late uninucleate and binucleate developmental stages of microspores have higher callus induction efficiency than those containing the microspore mother cell or tetrad stages. Calli derived from the uninucleate stage generally displayed more favorable visual characteristics, such as a compact texture and lighter coloration, compared with other stages. However, these observations were based on visual classification and were not directly quantified. Therefore, while such traits may be associated with actively dividing tissues, their relationship with regenerative potential should be interpreted cautiously in the absence of direct quantitative validation. The stage-dependent response is consistent with findings in other species. For example, anther cultures of kenaf [40] and bitter melon [41] have also shown maximal callus induction during the uninucleate stage. At this phase, microspores have not yet matured into pollen grains; they possess dense cytoplasm, relatively thin cell walls, and substantial nutrient and energy reserves. These characteristics increase their responsiveness to exogenous hormonal signals in the culture medium and promote the initiation of embryogenic pathways [42,43]. The rapid onset of callus induction and multiplication within three weeks in uninucleate anthers further highlights their sensitivity to the physiological state.

In the rubber tree, embryogenic callus was induced from the anther wall rather than from microspores, underscoring the strong influence of the microspore developmental stage on somatic embryogenesis. This observation suggests a functional relationship between microspore development and the embryogenic potential of adjacent somatic tissues in the anther wall, although the cellular mechanisms remain unclear [44]. Since these mechanisms were not experimentally investigated in the present study, they should be considered as hypotheses. The developmental status of microspores may influence the embryogenic competence of surrounding somatic cells through specific signaling molecules or physiological cues. Based on previous studies, this effect could involve a microspore-mediated intercellular signaling network in which metabolically active microspores at the uninucleate stage may release signaling compounds, including phytohormones, Ca^2+^, and soluble sugars [45,46,47]. Together with in vitro culture conditions and the physiological status of the tissues, these factors may help establish a microenvironment favorable for somatic cell regeneration. In addition, the tapetum, which closely interacts with developing microspores and provides nutrients and developmental signals, may also participate in somatic dedifferentiation through coordinated signaling exchanges [48,49].

In contrast, anthers excised at earlier developmental stages showed a slow, weak response, suggesting that their somatic tissues may have a reduced capacity to perceive or efficiently relay the inductive cues supplied by the culture medium. These findings are according to the previous reports, which found that the uninucleate stage is an important developmental window characterized by enhanced metabolic activity and increased cellular totipotency [50]. During this phase, the physiological and biochemical conditions appear to be particularly conducive to cellular reprogramming, thereby enhancing induction efficiency [23]. Histological analyses revealed that callus formation and the subsequent development of embryogenic structures originated from diploid somatic tissues of the anther wall, including the epidermis, endothecium, connective tissue, and filament. At no stage of development was direct embryogenesis from microspores observed. These findings are in line with previous reports in *Hevea brasiliensis* and other woody species, where intact anther cultures predominantly yield diploid regenerants derived from somatic tissues rather than haploid microspores [51,52,53]. Plant regeneration was also confined to embryos derived from uninucleate-stage anthers, with the early uninucleate stage producing the highest regeneration rate. Although binucleate-stage anthers generated some cotyledonary embryos, these failed to develop into plantlets, suggesting reduced developmental stability or physiological vigor at this stage.

## 4. Materials and Methods

### 4.1. Plant Material

The clonal rubber tree (*Hevea brasiliensis*) cultivar Reyan-73397 was used as the primary experimental material. Flowering shoots were collected from healthy, mature trees cultivated at the experimental field of the Rubber Research Institute, Chinese Academy of Tropical Agricultural Sciences, Danzhou, Hainan Province, China. Sampling was conducted during the early peak flowering periods in April, May, and July 2025, with well-developed inflorescences collected between 08:00 and 10:00, immediately transferred to insulated ice boxes, and then transported to the laboratory for further processing.

### 4.2. Procedures

#### 4.2.1. Analysis of Bud Morphology and Microspore Development

Fresh flower buds were randomly selected and analyzed using a stereomicroscope (Nikon SMZ7425T stereomicroscope; Nikon Corporation, Tokyo, Japan.). The color of the buds was visually noted, and their length and width were measured with image analysis. In this study, bud length was used as a morphological marker to estimate the microspore developmental stage. Based on cytological observations, provisional bud-length ranges were determined for each stage: microspore mother cell (1.4–1.8 mm), tetrad (1.8–2.2 mm), early uninucleate (2.2–2.6 mm), late uninucleate (2.6–2.9 mm), binucleate (2.9–3.2 mm), and mature pollen (>3.3 mm). These ranges served as reference criteria for selecting stage-specific buds in subsequent experiments. For stage confirmation during sampling, whole anthers were excised and observed under a light microscope. Multiple visual fields were examined per anther, and the bud was assigned to the developmental stage represented by the most dominant microspore population, following established criteria for rubber tree microsporogenesis [38,54]. Buds with mixed stages and no clearly dominant population were regarded as transitional and excluded from the study. If staging remained ambiguous after re-examining multiple buds of the same length, the sample was discarded to ensure classification accuracy. To verify these classifications, a second sampling was conducted using the same plant population. For each developmental stage, 10 buds within the specified length range were randomly collected per replicate, with 3 independent replicates, yielding a total of 30 buds per stage per treatment.

#### 4.2.2. Bud Fixation

The collected buds were dissected to reveal the anthers, and the color of the anthers was recorded. The stamens were removed and fixed in Carnoy’s (ethanol: glacial acetic acid; 3:1, *v*/*v*) solution for 12–16 h at room temperature. After fixation, the samples were rinsed three times with 75% ethanol and stored in 75% ethanol at 4 °C for cytological analysis.

#### 4.2.3. Cytological Analysis

Anthers were placed on clean glass slides, and the locules were opened longitudinally using a sterile scalpel. One to two drops of modified Carbol fuchsin stain (Solarbio, Beijing, China) were added to the sample at room temperature for 8–10 min. Excess stain was removed, glycerin was applied as a mounting medium, and the slides were covered with a coverslip. Gentle tapping was used to spread the tissue into a thin layer of cells. The prepared slides were examined under a light microscope (ZEISS Imager.Z2 microscope; Carl Zeiss Microscopy GmbH, Jena, Germany), and images were captured using the imaging software (ZEN 3.9 software; Carl Zeiss Microscopy GmbH, Jena, Germany). Because microspore development in rubber tree anthers is asynchronous, multiple locules from the same bud and several microscopic fields were examined. Each bud was assigned to the predominant developmental stage observed. Buds with a single clearly dominant developmental stage were used for culture; those with overlapping or mixed stages were regarded as transitional and excluded from subsequent experiments.

#### 4.2.4. Histological Analysis

To confirm the morphological characteristics of flower buds by histological analysis, three representative buds from each developmental stage were preserved in FAA (Formaldehyde, Acetic Acid, and Ethanol) solution (70% ethanol) for 24 h. To determine the cellular origin and developmental progression of anther-derived callus, swelling anthers from each developmental stage were collected at 21, 28, and 35 days and fixed in FAA solution. Following this, the samples were embedded in paraffin, sectioned at 8 μm, and stained with safranin and fast green by using the modified method recently used by Moreno-Sanz [54]. Following fixation, the calli were removed from the fixative and dehydrated. Dehydration was performed sequentially with increasing ethanol concentrations: 75% ethanol for 4 h, 85% ethanol for 2 h, 90% ethanol for 2 h, and 95% ethanol for 1 h, followed by two treatments with absolute ethanol for 30 min each. The samples were then treated with benzene for 5–10 min and cleared with two successive immersions in xylene, each lasting 5–10 min. After clearing, the calli were infiltrated with molten paraffin wax at 65 °C through three consecutive steps to ensure complete penetration. For embedding, molten paraffin was dispensed into embedding molds. Before the wax solidified, the dehydrated calli were removed from the cassettes, carefully oriented in embedding frames according to the desired sectioning plane, and properly labeled. The molds were placed in an embedding system to allow the paraffin to harden. The resulting paraffin blocks were stored at −20 °C to facilitate trimming and handling. For section preparation, the blocks were cooled again at −20 °C and sectioned using a microtome to obtain slices approximately 8 µm thick. The sections were floated on a 40 °C water bath to flatten them and then mounted onto glass slides. The slides were dried in an oven at 60 °C to remove moisture and excess paraffin, followed by cooling to room temperature. For histological analysis, sections were stained using the Safranin O–Fast Green method. Slides were first dewaxed in an environmentally friendly transparent solution for 20 min with two changes and then rehydrated through absolute ethanol I and II (5 min each), followed by 75% ethanol for 5 min and rinsing with tap water. Sections were stained with Safranin O for about 2 h and washed with tap water. Differentiation was performed briefly in 50% and 70% ethanol and then treated with 80% ethanol. Counterstaining with Fast Green was carried out for 6–20 s. Finally, the slides were dehydrated through three changes in absolute ethanol, cleared in xylene for 5 min, and permanently mounted with neutral gum for microscopic observation. Imaging was performed by Wuhan Saiweier Biotechnology Co., Ltd. (China), and photographs were analyzed with CaseViewer 2.4 software (Servicebio Technology Co., Ltd., Wuhan, China), focusing on callus initiation sites and developmental changes in the respective anthers. The developmental stages of microspores and the structures of the anther wall observed in histological sections were validated by comparing them with corresponding features of prepared slides, confirming accurate stage identification.

#### 4.2.5. Callus Induction from Anthers at Different Developmental Stages

Selected and separated flower buds at the microspore mother cell, tetrad, early uninucleate, late uninucleate, and binucleate stages were surface-sterilized with 75% ethanol for 1 min, rinsed three times with autoclaved distilled water, treated with 0.1% HgCl_2_ for 10 min, and washed 5–6 times with autoclaved distilled water. Sterilized buds were dissected under a stereomicroscope to obtain intact stamens, which were cultured on anther callus induction medium. The induction medium on MS-based callogenesis medium (MSC) [55] consisted of modified MS (composition detailed in Supplementary S1) + 2,4-D (2,4-dichlorophenoxyacetic acid), NAA (Naphthalene Acetic Acid) and Kt (Kinetin) at 1.5 mg/L, supplemented with 0.3 g L^−1^ asparagine and 50% coconut water, adjusted to pH 5.8 and autoclaved at 121 °C for 20 min. For each developmental stage, 21 culture tubes (7 tubes × 3 replicates) were prepared, with four anthers per tube (28 anthers per replicate). Cultures were maintained in dark conditions at 25 ± 2 °C. Callus induction was evaluated after 35 days.

To quantitatively assess callus development, a visual scoring system ranging from 0 to 3 was established based on growth performance during culture:

Score 0: Anther completely browned, with no callus formation;

Score 1: Anther viable with slight anther sac swelling, but no visible callus;

Score 2: Distinct callus formed, but with limited growth (callus area < 25 mm^2^);

Score 3: Vigorous callus with abundant growth (callus area ≥ 25 mm^2^).

**Figure 6:**
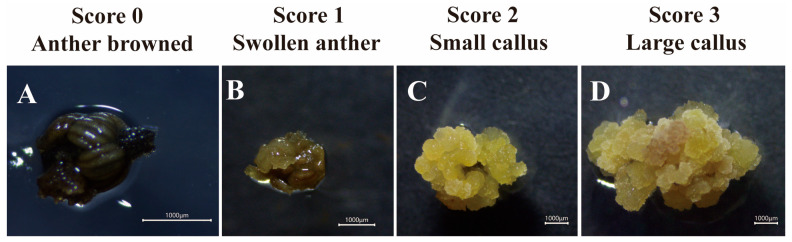
Anther-based callus scoring system for *Hevea* clone 73397. (**A**) Browning of the anther, indicating tissue death, was assigned a score of 0. (**B**) Swollen anthers showing initial callus induction were scored as 1. (**C**) Anthers producing a small quantity of callus were given a score of 2, representing callus proliferation. (**D**) Anthers with a large amount of callus formation were assigned a score of 3, indicating active callus multiplication.

This scoring scale is illustrated in Figure 6. All anthers within each replicate were individually scored, and the mean callus score per replicate was calculated to compare the influence of microspore developmental stage on callus growth vigor.

Callus induction frequency (%) = (number of anthers producing callus/total anthers cultured) × 100.

Total score _R_ =∑ (Callus grade × Score), R is a replicate, and R = 1,2,3.

Average score = (Total score_1_ + Total score_2_ + Total score_3_)/3

#### 4.2.6. Embryo Conversion and Plant Regeneration

Induced callus from responsive anthers of each developmental stage was transferred to embryo induction media supplemented with 2,4-D, after 45 days of callus induction, and incubated at 25 °C under dark conditions. Data on embryo inductions were evaluated after 50 days of culture. The induction medium was based on MS-based embryogenesis medium (MSE) [55], lacking abscisic acid, and supplemented with 0.06 mg 2,4-D (0.06 mg/L) + Kt (3.0 mg/L) + BAP; 6-Benzylaminopurine (1.0 mg/L) + GA_3_; Gibberellic acid (0.5 mg/L) + activated charcoal (1.0 g/L) + coconut water (50 mL/L) with pH adjusted to 5.8 before autoclaving. For regeneration of induced cotyledonary embryos, they were transferred to MS-based plant regeneration medium (MSR) [55] consisting of modified MS with Kt (0.5 mg/L) + IAA; indole-3-acetic acid (0.2 mg/L) + GA_3_ (0.03 mg/L) + sucrose (5%) + coconut water (50 mL/L) + activated charcoal (1.0 mg/L) + Phytagel (1.0 g/L) and cultured under control photoperiod conditions (16:8; light: dark) at 25 ± 2 °C with a light intensity of 3500–3800 lux. Regeneration data was evaluated after 40 days of culture.

Embryogenic callus rate (%) = (number of embryogenic calli/total number of calli cultured) × 100.

Plant regeneration rate (%) = (number of regenerated plantlets/number of cotyledonary embryos transferred) × 100.

### 4.3. Statistical Analysis

All data were compiled in Microsoft Excel 2019 and statistically analyzed using IBM SPSS Statistics version 27. Treatment effects were evaluated by one-way analysis of variance (ANOVA). When significant differences were observed, means were separated using Duncan’s new multiple range test at a significance level of *p* < 0.05. Figures were generated using Adobe Illustrator 2020.

## 5. Conclusions

This study examined the relationship between external bud morphology and internal microspore developmental stages in the rubber tree clone ‘Reyan 73397’ under the specific experimental conditions tested. Among the six cytologically defined stages, the uninucleate phase—particularly the early- to late-uninucleate stage—showed the highest responsiveness for callus induction, somatic embryo formation, and subsequent plantlet regeneration. In contrast, anthers at the microspore mother cell and tetrad stages exhibited little to no embryogenic response, whereas those at the binucleate stage displayed moderate callus induction with comparatively lower regeneration efficiency. These findings indicate that the microspore developmental stage plays an important role in anther-derived somatic embryogenesis in *Hevea brasiliensis*, within the context of the genotype studied. Histological observations were consistent with a somatic origin of both callus and somatic embryos, with tissues appearing to arise from diploid anther wall components, including the epidermis, endothecium, connective tissue, and filament. However, as a complete ontogenetic sequence was not established, these results should be interpreted as suggestive rather than definitive evidence of tissue origin. The results further indicate that intact anther culture in this clone predominantly favors somatic embryogenesis over haploid production, while still serving as a useful approach for diploid plant regeneration. A clear association was also observed between internal microspore developmental stages and external floral characteristics. Traits such as bud length, transverse diameter, and bud or anther color appeared to be practical morphological indicators for identifying the optimal developmental stage, although further validation with additional genotypes and statistical support would strengthen these observations. Moreover, histological examination of the later stages of embryo development was not conducted in this study. Future research incorporating detailed histological analyses would provide deeper insights into the progression and origin of embryo development. Such studies would strengthen the understanding of embryogenic pathways and contribute to the optimization of protocols for improved embryo induction and plant regeneration.

In summary, buds with a transverse diameter of 1.42–1.57 mm and a green to greenish-yellow color were strongly associated with the uninucleate microspore stage, which showed the highest embryogenic potential. Cytological verification combined with rapid and nondestructive morphological screening provides a practical and efficient method for large-scale explant selection. This study identifies a precise developmental window that maximizes anther culture efficiency in rubber trees. It also clarifies the somatic origin of embryogenic tissues and establishes reliable morphological and cytological criteria for selecting suitable explants in future research. These findings provide a robust experimental foundation for optimizing in vitro regeneration systems and advancing biotechnological strategies in future rubber tree breeding programs.

## Figures and Tables

**Table 1:** Developmental stage classification and morphological characteristics of flower buds.

Floral Development Stage	Length (mm)		Width (mm)		Morphological Character	
	Range	Average	Range	Average	Flower Bud (Color)	Anther (Texture)
Microspore Mother Cell	1.4–1.8	1.68 ± 0.09 ^f^	1.01–1.24	1.14 ± 0.09 ^d^	Light green	Tender and translucent
Tetrad	1.8–2.2	2.02 ± 0.12 ^e^	1.09–1.39	1.25 ± 0.08 ^d^	Light green	Gel-like and milky white
Early Uninucleate	2.2–2.6	2.34 ± 0.10 ^d^	1.28–1.57	1.42 ± 0.08 ^c^	Green	Slightly firm and creamy yellow
Late Uninucleate	2.6–2.9	2.74 ± 0.09 ^c^	1.32–1.77	1.57 ± 0.09 ^b^	Greenish- yellow	Pliable and pale yellow
Binucleate	2.9–3.2	3.04 ± 0.08 ^b^	1.45–1.80	1.65 ± 0.06 ^b^	Yellowish-green	Semi-rigid yet flexible, light yellow
Maturity	>3.3	3.46 ± 0.13 ^a^	1.71–2.43	1.97 ± 0.18 ^a^	Yellow	Not fully hardened, yellowish-white

Note: Letters a–f indicate significant differences, where *p* < 0.05.

**Table 2:** Callus induction from anthers containing microspores at different stages of development, after 35 days of inoculation.

Floral Development Stage	Number of Inoculated Anthers/Piece	Number of Callus Formed/Piece	Callus Induction Rate (%)	Average Score	The Speed of Callus Initiation
Microspore mother cell	28	6.67 ± 0.58	23.81 ^c^	11.67 ± 5.86 ^c^	4 weeks
Tetrad	28	9.00 ± 1.00	32.14 ^b^	33.00 ± 10.44 ^b^	4 weeks
Early Uninucleate	28	28.00 ± 0.00	100.00 ^a^	63.33 ± 3.06 ^a^	3 weeks
Late Uninucleate	28	27.67 ± 0.58	98.81 ^a^	64.33 ± 1.15 ^a^	3 weeks
Binucleate	28	27.33 ± 0.58	97.62 ^a^	63.33 ± 1.15 ^a^	3 weeks

Note: Letters a–c indicate significant differences at (*p* < 0.05).

**Table 3:** Somatic embryo induction and plant regeneration from calli derived from microspores at different developmental stages.

Floral Development Stage	Number of Inoculated Calli (Mass)	After 50 Days			After 35 Days	
		Number of Developed Embryoids (Units)	Embryogenic Callus Rate (%)	Number of Cotyledonary Embryos	Number of Regenerated Plantlets	Regeneration Efficiency (%)
Microspore mother cell	6.67 ± 0.58	0 ± 0.00	0.00 ^c^	0	0	0.00
Tetrad	9.00 ± 1.00	0 ± 0.00	0.00 ^c^	0	0	0.00
Early Uninucleate	28.00 ± 0.00	25 ± 0.67	29.76 ^a^	8	4	50.00
Late Uninucleate	27.67 ± 0.58	16 ± 1.67	19.28 ^ab^	6	1	16.67
Binucleate	27.33 ± 0.58	10 ± 1.86	12.20 ^bc^	3	0	0.00

Note: Letters a–c indicate significant differences at *p* < 0.05.

## Data Availability

All data are presented within the article.

## Supplementary Materials

The following supporting information can be downloaded at: https://www.mdpi.com/article/10.3390/plants15060973/s1, Supplementary S1: Information of the modified MS medium used in this study.

## Abbreviations

The following abbreviations are used in this manuscript:

MSC	MS-based callogenesis medium
MSE	MS-based embryogenesis medium
MSR	MS-based plant regeneration medium
MS	Murashige and Skoog 1962
2,4-D	2,4-dichlorophenoxyacetic acid
NAA	Naphthalene Acetic Acid
Kt	Kinetin
BAP	6-Benzylaminopurine
GA3	Gibberellic acid
IAA	Indole-3-acetic acid
