# Supplementary material for: Defining the Optimal Microspore Developmental Window for Efficient Anther-Derived Somatic Embryogenesis in Rubber Tree (Hevea brasiliensis)
Source: Plants (Basel). 2026 Mar 21;15(6):973. doi: 10.3390/plants15060973 (PMC13030732; doi:10.3390/plants15060973)
Supplement: Supplementary file 1 [file plants-15-00973-s001.zip › plants-4179376-supplementary.pdf]

In this study, a modified MS medium was used, and its stock solution formulation was compared with that of the standard MS medium (commercial MS) as shown in the following table. All MS I–IV stock solutions were prepared and stored at 4 ℃ in a refrigerator. Adjustments were made to the macronutrient, micronutrient, and vitamin components in the modified MS medium, with the specific differences as follows:

Modified MS I			MS I	
Macronutrients	Amount (g/l)20X		Macronutrients	Amount (g/l)20X
KNO3	38		KNO3	38
NH4NO3	33		NH4NO3	33
CaCl2	5.19		CaCl2.2H2O	8.8
MgSO4.7H2O	11.1		MgSO4.7H2O	7.4
KH2PO4	8.5		KH2PO4	3.4
Modified MS II			MS II	
Micronutrients	Amount (g/l)100X		Micronutrients	Amount (g/l)200X
KI	0.08		KI	0.166
H3BO3	1		H3BO3	1.24
MnSO4.H2O	3.031		MnSO4.4H2O	4.46
ZnSO4.7H2O	1		ZnSO4.7H2O	1.72
Na2MoO4.2H2O	0.025		Na2MoO4.2H2O	0.05
CuSO4.5H2O	0.025		CuSO4.5H2O	0.005
CoCl2.6H2O	0.0025		CoCl2.6H2O	0.005
Modified MS III			MS III	
Constituents	Amount (g/l)100X		Constituents	Amount (g/l)100X
FeSO4.7H2O	2.78		FeSO4.7H2O	2.78
Na2EDTA.2H2O	3.73		Na2EDTA.2H2O	3.73
Modified MS IV			MS IV	
Constituents	Amount (g/l) 100X		Constituents	Amount (g/l) 100X
Myo-inositol	10		Myo-inositol	10
ThiamineHCl (VB1)	0.05		ThiamineHCl	0.1
Pyridoxine HCl (VB6)	0.05		Pyridoxine HCl	0.5
Nicotinic acid (VB5)	0.5		Nicotinic acid	0.5
Glycine	0.2		Glycine	0.2
Folic acid	0.05	（Dissolve separately with 1 M NaOH solution.）		
D-Biotin	0.005			
